# Antibodies to SARS-CoV-2 and their potential for therapeutic passive immunization

**DOI:** 10.7554/eLife.57877

**Published:** 2020-06-23

**Authors:** PJ Klasse, John P Moore

**Affiliations:** Department of Microbiology and Immunology, Weill Cornell MedicineNew YorkUnited States; Attikon University HospitalGreece; Radboud University Medical CentreNetherlands

**Keywords:** COVID-19, SARS-CoV-2, antibodies, vaccine, plasma, therapy

## Abstract

We review aspects of the antibody response to SARS-CoV-2, the causative agent of the COVID-19 pandemic. The topics we cover are relevant to immunotherapy with plasma from recovered patients, monoclonal antibodies against the viral S-protein, and soluble forms of the receptor for the virus, angiotensin converting enzyme 2. The development of vaccines against SARS-CoV-2, an essential public health tool, will also be informed by an understanding of the antibody response in infected patients. Although virus-neutralizing antibodies are likely to protect, antibodies could potentially trigger immunopathogenic events in SARS-CoV-2-infected patients or enhance infection. An awareness of these possibilities may benefit clinicians and the developers of antibody-based therapies and vaccines.

## Introduction

Passive immunization with plasma from patients who have seroconverted to and recovered from infection with a pathogen has a long and generally successful history. It has been used extensively against influenza virus and on a small scale during the 1995 and 2014–2015 Ebola epidemics (Brown et al., 2018; Mupapa et al., 1999; Mair-Jenkins et al., 2015; Hung et al., 2011; Luke et al., 2006). Purified polyclonal (sometimes referred to as polyvalent) immunoglobulin (Ig) from convalescents has been administered prophylactically after exposure to infectious virus (Young, 2019). In recent years, highly specific and often broadly active neutralizing monoclonal antibodies (MAbs) have been developed against several viruses, as a more advanced substitute for patient plasma (Caskey et al., 2019; Corti et al., 2016; Corti et al., 2017; Walker and Burton, 2018; Wec et al., 2019; Zheng et al., 2020). These methods are now being considered for treating COVID-19, the disease caused by the SARS-CoV-2 coronavirus (Dhama et al., 2020; Jawhara, 2020; Ju et al., 2020; Zhou and Zhao, 2020; Accorsi et al., 2020; Bloch et al., 2020; Sullivan and Roback, 2020). Several reports describe apparent benefits, with no adverse side effects, when convalescent plasma was infused into patients with SARS-CoV-1 or SARS-CoV-2 infection (Table 1; Cheng et al., 2005; Yeh et al., 2005; Soo et al., 2004; Shen et al., 2020; Duan et al., 2020; Zhang et al., 2020; Ahn et al., 2020). The US Food and Drug Administration has recently approved plasma immunotherapy for this purpose, and has outlined safety criteria (https://www.fda.gov/vaccines-blood-biologics/investigational-new-drug-ind-or-device-exemption-ide-process-cber/recommendations-investigational-covid-19-convalescent-plasma). To determine the efficacy of convalescent plasma to treat COVID-19, the FDA has called for randomized clinical trials and encouraged investigational new drug applications (Bloch et al., 2020; Sullivan and Roback, 2020). Here, we review aspects of the antibody response to SARS-CoV-2, which may be relevant to immunotherapy with plasma or MAbs. A major goal of viral vaccine development is the induction of strong and broadly active neutralizing antibodies (NAbs), and that goal applies also to SARS-CoV-2 (Dhama et al., 2020; Graham, 2020; Amanat and Krammer, 2020). The development of vaccines, an essential public health tool, will also be informed by an understanding of the antibody response during SARS-CoV-2 infection.

**Table 1.** Passive immunization with convalescent plasma (CP) during SARS-CoV-1 and SARS-CoV-2 infection.

Reference	Virus	Antibody source	Number of patients	Efficacy	Safety
Cheng et al., 2005	SARS-CoV-1	CP 160–640 ml Seropositive titer range: 160–2,560	80 patients with SARS	Better outcome with plasma before than after day 14	No immediate adverse effects
Yeh et al., 2005	SARS-CoV-1	CP 500 ml IF IgG titer >640	3 hospital workers with SARS	Drop within 24 hr in viral load from ~ 10^5^ to < 1 RNA copies/ml	No significant side effects
Soo et al., 2004	SARS-CoV-1	CP Ab titers not measured	19 (plasma) vs. 21 (methylprednisolone) SARS patients	Faster release, lower mortality with plasma than comparator	No immediate adverse effects
Shen et al., 2020	SARS-CoV-2	CP 400 ml Ab binding >1000 NAb > 40	5 COVID-19 patients	Reduced viral load, clinical improvement Release of 3/5	None reported
Duan et al., 2020	SARS-CoV-2	CP 200 ml NAb > 640	10 COVID-19 patients	Virus undetectable in 7/10 Varying clinical, laboratory, radiological improvements	No adverse effects observed
Zhang et al., 2020	SARS-CoV-2	CP 200–2,400 ml Ab not measured	4 COVID-19 patients	Negative PCR Pulmonological improvements Discharge of 3/4	No adverse effects observed
Ahn et al., 2020	SARS-CoV-2	CP 2 × 250 ml Binding IgG detected by ELISA	2 COVID-19 patients	Reduced sputum viral load Radiological and clinical improvements	No adverse effects observed

Assays are now available for detecting IgA, IgM, and IgG specific for SARS-CoV-2 in patient serum, that is to demonstrate seroconversion, and also for detecting NAbs (Amanat et al., 2020; Wu et al., 2020). These techniques are rapidly evolving, and additional information on the antibody response to CoV-2 infection is emerging almost daily. Analyses of how long predictably protective titers are maintained are still lacking. They will be a priority once enough time has elapsed to allow long-term studies.

The natural history of COVID-19 and some lessons from infections with the previous SARS coronavirus (SARS-CoV-1) and the more distantly related MERS-CoV, including animal model studies, do raise some concerns about NAb-based therapies and vaccines, warranting careful surveillance by clinicians during human trials. Furthermore, certain approaches may minimize risks while preserving the benefits of passive immunization for curing COVID-19.

### Antibody-mediated neutralization of SARS-CoV-2

The entry of SARS-CoV-2 into cells is initiated by the interaction of the receptor-binding domain (RBD) of the viral Spike (S) glycoprotein with the angiotensin converting enzyme-2 (ACE2), which acts as a receptor for the virus on the target cell surface (Hoffmann et al., 2020; Ou et al., 2020). The most potent NAbs are directed to the RBD and some may act by simply competing with the receptor for binding to the S-protein. Antibodies to SARS-CoV-1 and MERS-CoV generally do not cross-neutralize SARS-CoV-2; although cross-reactive antibodies are frequently detected in S-protein ELISA (Ju et al., 2020; Wu et al., 2020; Ou et al., 2020; Chen et al., 2005; Quinlan et al., 2020; Wrapp et al., 2020). Recently, however, the S-protein-specific NAb S309, isolated from memory B cells of a patient who had recovered from CoV-1 infection in 2003, was shown to neutralize both SARS-CoV-1 and −2 potently by ligating the RBD. Cryo-electron microscopy and binding assays demonstrated that the conserved S309 epitope comprises glycans and that in spite of the specificity of the Mab for the RBD, it does not interfere with ACE2 binding (Pinto et al., 2020).

The neutralizing potency of antibodies against the RBD may be determined not only by their own affinity for the S-protein but also by the affinity of the latter for ACE2, at least when they act by a competitive mechanism (Ju et al., 2020). In this context, it is notable that the SARS-CoV-2 S-protein has a 4–20-fold higher affinity for ACE2 than its counterpart from SARS-CoV-1 (35, 37). Although most NAbs to SARS-CoV-1 and −2 are directed to the RBD (Pinto et al., 2020; Coughlin et al., 2007; Greenough et al., 2005; Sui et al., 2004; ter Meulen et al., 2006; van den Brink et al., 2005; Zhu et al., 2007), some antibodies that recognize the SARS-CoV-1 S2 fragment can also neutralize (Duan et al., 2005; Elshabrawy et al., 2012). In addition, antibodies to the ectodomain of another surface-exposed SARS-CoV-1 protein, Orf3a, are also reported to have neutralizing activity, while antibodies to the M and E proteins can potentiate neutralization (Akerström et al., 2006; Buchholz et al., 2004). Whether SARS-CoV-2 is similar to SARS-CoV-1 in all these respects remains to be determined. Nonetheless, passive and active immunization approaches to COVID-19 are generally focused on NAbs against the S1-protein.

### The kinetics of NAb and other antibody responses in SARS-CoV infection

The information on the antibody responses elicited in COVID-19 patients is growing fast, but none is yet available about the longevity of the immunity. Data on SARS-CoV-1 infection may, however, be informative in that respect. Surprisingly, the NAb response in patients who later succumbed to the infection has been found to be faster than in those who recovered; in the patients who later died, the titers had peaked around day 15 after the onset of symptoms, whereas similar titers and extents of neutralization were reached only after day 20 in the patients who recovered (Zhang et al., 2006; Ho et al., 2005). The NAb titers in the moribund patients declined or disappeared after the early rise, as their conditions deteriorated towards death (Zhang et al., 2006). It is unknown whether this titer loss reflects an inability to produce antibodies due to lymphocyte losses. As NAb titers rise, however, viral loads decline, presumably because virus replication diminishes (Wölfel et al., 2020; To et al., 2020). These findings do not exclude the possibility that the initial viral loads, before NAbs emerged, were particularly high and stimulated stronger and earlier antibody responses in the patients who subsequently became most severely affected.

In plasma collected from 175 patients who had recovered from mild COVID-19, NAb and S-binding-antibody titers correlated positively with age and CRP (C-reactive protein) levels, but negatively with lymphocyte counts; and the NAbs did not cross-neutralize SARS-CoV-1 (Wu et al., 2020). Since no severe cases were included and viral loads were not monitored, it is unclear what promoted the NAb responses within the patient cohort in which antibody titers, age (range 16–85 years), lymphopenia, and inflammation were associated. The positive correlation between NAb responses and age contrasts with a general decline in the vigor of new B-cell responses in the elderly (Siegrist and Aspinall, 2009), but raises the question whether the pre-response viral load was correlated with age, which in turn correlates with disease severity. Other studies have shown higher binding-antibody titers to the nucleocapsid protein N in patients who recovered than in those who did not (Wu et al., 2020; Leung et al., 2004). Such antibodies to the N-protein, which is internal and thus not exposed on the surface of the virion, completely lack neutralizing capacity but their production might reflect the strength of T-helper cell responses (Klasse et al., 2012).

### NAb immunotherapy against SARS-CoV-1 and SARS-CoV-2

Will passive immunization with plasma from convalescent patients be beneficial for treating COVID-19? Anti-S antibodies seem to protect against lethal CoV challenge and clear the virus in mice and ferrets (Du et al., 2008a; Du et al., 2007; Du et al., 2008b; Fett et al., 2013). In a small experiment, SARS-CoV-2 infection reportedly protected against a second challenge of macaques, which was attributed to the development of protective antibodies (Bao et al., 2020). The outcome of human clinical trials will, of course, outweigh animal-model experiments.

No significant adverse reactions were noted when plasma with high NAb titers were given to SARS-CoV-1 patients; benefits such as lower viral loads and earlier release from hospital were noted in retrospective analyses (Cheng et al., 2005; Yeh et al., 2005; Soo et al., 2004; Table 1). Recently, five critically ill COVID-19 patients were transfused at 10–22 days post-admission with a pool of plasma derived from five convalescent patients; the RBD-binding antibody endpoint titers in ELISA were >1000, and the neutralization endpoint titers were >40 (Shen et al., 2020). All the patients (36–65 years; three male, two female) were receiving mechanical ventilation. After plasma transfusion, body temperatures normalized, while organ-failure and respiratory-function scores improved to various extents. Nasopharyngeal viral loads decreased and became undetectable within 12 days in all five patients, while SARS-CoV-2 ELISA and NAb titers increased, reflecting the antibody-content of the transfused plasma. Thus, in this preliminary and necessarily uncontrolled case series of five critically ill COVID-19 patients with acute respiratory distress syndrome (ARDS), the transfusion of NAb-containing convalescent plasma was associated with improved clinical status (Shen et al., 2020). A subsequent larger study yielded similar results: ten patients with severe COVID-19 received 200 mL of convalescent plasma obtained from recently recovered donors with NAb inhibitory-dilution factors > 640. Three days later, clinical, pulmonary-radiological, and laboratory parameters were improved, the latter including oxyhemoglobin saturation, lymphocyte counts, and C-reactive protein levels; viral loads in serum became undetectable in seven patients (Duan et al., 2020; see also additional smaller studies in Table 1). Overall, these studies showed plasma transfusion to be well tolerated. Although beneficial effects were reported, they could not be proven because the studies were not controlled and included other antiviral interventions.

### Can antibodies contribute to SARS pathogenesis?

Strategies for passive and active immunization to combat and prevent SARS-CoV-2 infection should take into account the pathogenesis of COVID-19, which can lead to death. The inflammatory response to SARS-CoV-2 is thought to drive or at least exacerbate the disease process, particularly during the second week after infection becomes symptomatic. How may these immune responses that modulate pathogenesis be affected by NAbs?

The lethal coronaviruses cause fatal acute lung injury (ALI) by driving hypercytokinemia and aggressive inflammation through incompletely understood mechanisms. In macaque models of SARS-CoV-1 infection and passive or active immunization, IgG specific for the S-protein was reported to exacerbate ALI by counteracting inflammation-resolving responses, abrogating wound-healing, promoting monocyte chemoattractant peptide-1 (MCP-1) and interleukin-8 (IL-8) production, and increasing proinflammatory monocyte and macrophage recruitment (Liu et al., 2019). Likewise, in human patients who died of SARS-CoV-1 infection, pulmonary proinflammatory macrophages accumulated in the lungs, whereas wound-healing macrophages were absent (Liu et al., 2019). Moreover, two observations noted above raise questions about the causal relationship between antibodies and severity of infection: NAb responses were faster in the patients who later died than in those who recovered (Zhang et al., 2006; Ho et al., 2005; Liu et al., 2019), and older patients who had recovered from mild COVID-19, had significantly stronger NAb and S-protein-binding antibody responses than younger ones, whereas higher age is a major risk factor for lethal COVID-19 (Wu et al., 2020).

In vitro, sera from subsequently deceased patients enhanced SARS-CoV-1 induced MCP-1 and IL-8 production by human monocyte–derived wound-healing macrophages, whereas blockade of the FcγR receptor reduced these effects (Liu et al., 2019). One must be prudent when extrapolating from a macaque model of SARS-CoV-1 infection to human COVID-19 patients, but the antibody response to these lethal coronaviruses might play a role in disease progression, perhaps by formation of immune complexes, and by promoting macrophage infiltration and sustained inflammation. We hypothesize that there may be a causal link between seroconversion and the rapid deterioration that can take place in the second week after the first symptoms, but this remains to be established.

Other reports suggest that anti-S and other CoV-specific antibodies have pathogenic effects in animal models. Thus, multiple CoV vaccines were associated with an increase in eosinophilic proinflammatory pulmonary responses upon challenge of the immunized animals (Bolles et al., 2011; Honda-Okubo et al., 2015; Iwata-Yoshikawa et al., 2014). Previous SARS-CoV-1 infection limited virus replication in African green monkeys but not lung inflammation, when the animals were re-challenged with the same virus (Clay et al., 2012). It has not been determined which factors, such as viral dose and the extent of the innate and adaptive immune responses, yield these problematic effects. A particularly important knowledge gap is whether certain specificities and other properties of antibodies are responsible.

Pre-existing serum antibodies against influenza antigens were consistently associated with severe illness in patients during the 2009 influenza A H1N1 pandemic (To et al., 2012; Monsalvo et al., 2011). Of note is that those antibodies did not neutralize influenza virus (To et al., 2012) and that immune complex formation was implicated as a pathogenic trigger (Monsalvo et al., 2011). Whether these observations are linked to the findings reported by Liu et al. remains to be seen (Liu et al., 2019).

### Antibody-dependent enhancement of infection (ADE)

Antibodies can also exacerbate viral infection by different mechanisms that have long been described (Halstead, 1982). In the vaccine context, infection by alpha- and flaviviruses (such as Dengue and Zika viruses) is enhanced when the antibody occupancy on the virion-surface epitopes falls below a critical threshold (Pierson and Diamond, 2015). This is the stoichiometric condition of an Fc-receptor-dependent form of ADE: the same antibodies that mediate ADE can neutralize and protect at higher occupancies on virions; alternatively, non-NAbs binding to epitopes exposed on the virion surface to antigens that are not functional for mediating entry may confer ADE (Pierson and Diamond, 2015; Klasse, 2014). The in vitro observations of ADE seem to account for the unfortunate outcome of recent Dengue vaccine trials with examples of worsened disease post-infection (Hurtado‐Monzón et al., 2020). ADE has been reported in the coronavirus literature, although most studies do not suggest that it will be as problematic as for alpha- and flaviviruses (Jaume et al., 2011; Kam et al., 2007; Peeples, 2020; Wan et al., 2020; Wang et al., 2014; Wang et al., 2016; Diamond and Pierson, 2020; de Alwis et al., 2020; Burton and Walker, 2020). An exception is a study of vaccinia-vectored immunization of kittens with the S protein of the coronavirus feline infectious peritonitis virus. The vaccine induced NAbs poorly. After challenge with the infectious virus, deaths occurred sooner in the S-protein-vaccine group than in the vaccinia-only control group (Diamond and Pierson, 2020; Vennema et al., 1990).

Particular problems of ADE could arise in the face of an ongoing epidemic through NAbs at sub-protective levels, whether after incomplete vaccination courses or with poor and rapidly declining vaccine responses, as well as after passive immunization because of insufficient efficacy of NAbs in plasma or in purified polyclonal Ig and of MAbs.

One recent report described an unusual mechanism of MERS-CoV-infection enhancement in vitro, whereby the antibody binding to the S protein RBD promoted endocytic uptake by engaging with an Fc-receptor and triggered fusion by inducing a conformational change (Jaume et al., 2011). It augurs well for vaccine development, however, that a SARS-CoV-2 RBD used as an immunogen elicited strong NAb responses in rats, without any ADE (Quinlan et al., 2020). These topics will, no doubt, be investigated thoroughly as much-needed SARS-CoV-2 vaccines undergo pre-clinical and clinical testing.

### Possible improvements to immunotherapy

How could therapeutic interventions be improved so as to preserve the capacity of the infused NAbs to reduce virus replication while preventing the possible induction of fatal ALI through promotion of IL-8 and MCP-1 production and inflammatory macrophage accumulation? One precaution would be to administer NAbs with Fc deletions. In principle, this could be accomplished by enzymatic treatment of polyclonal IgGs purified from plasma to generate bivalent F(ab’)two fragments. But in practice this would probably be too onerous. More feasible is the genetic engineering of neutralizing MAbs to eliminate the ability of the Fc-domains to bind activating FcR:s. Although such mutations would also eliminate potentially beneficial Fc-mediated effects such as ADCC, there is no evidence that these effector functions play a role in reducing viral load. For that goal, virus neutralization may be necessary and sufficient, at least during the COVID-19 acute phase.

An alternative neutralizing intervention, which eliminates some risks associated with polyclonal and monoclonal antibodies, is the use of a soluble, recombinant form of the ACE2 receptor, which is potent (nM range) and effective (depending on target cells) at blocking SARS-CoV-2 infection in vitro (Lei et al., 2020). Since the SARS-CoV-2 S-protein has a 4–20-fold higher affinity than the SARS-CoV-1 S-protein for ACE2, it may be more sensitive to this particular intervention, at least under some conditions of infection (Wrapp et al., 2020; Walls et al., 2020). Other advantages of these constructs are their potency and potential breadth of action against new viral variants. But if Fc-receptor ligation is pathogenic (Liu et al., 2019), methods of increasing avidity other than fusing the soluble receptor to the Fc portion of IgG could be explored. The effects on angiotensin activation and its pharmacological inhibition may also need to be evaluated (Hoffmann et al., 2020; Aronson and Ferner, 2020).

### Conclusions

Plasma infusion as therapy for COVID-19 is a stop-gap measure that is now being used in a medical emergency. Within the next year, effective drugs are likely to emerge, and they may well include highly potent and specific MAbs to the SARS-CoV-2 S-protein. Animal experiments, particularly in macaques, will be valuable for comparing the capacity of different monoclonal and polyclonal antibodies, including combinations, or of recombinant receptor mimics, to clear SARS-CoV-2 infection. Ideally, the intervention should permit or even promote the emergence of favorable innate responses and the resolution of inflammation (Nathan and Ding, 2010). Given the urgency of the COVID-19 pandemic, however, it may be impossible to perform such studies before human trials. Furthermore, differences in Fc-receptor biology may invalidate some extrapolations of antibody effects from macaques to humans (Bournazos and Ravetch, 2017). In these circumstances, an awareness of what has occurred in other viral infections, particularly with SARS-CoV-1, as well as what is now being published on SARS-CoV-2, may guide both treatment strategies and the development of antibody-based vaccines (Peeples, 2020; Tseng et al., 2012; Agrawal et al., 2016). Prospective or retrospective analyses of how the binding-antibody and NAb titers of transfused plasmas are associated with clinical improvements should also guide both MAb-based therapies and vaccine evaluation. If apparently antibody-mediated adverse events do occur, they too should help to improve these important public health measures against the COVID-19 pandemic.
